# Role of Androgen Receptor in Progression of LNCaP Prostate Cancer Cells from G_1_ to S Phase

**DOI:** 10.1371/journal.pone.0056692

**Published:** 2013-02-20

**Authors:** Shalini Murthy, Min Wu, V. Uma Bai, Zizheng Hou, Mani Menon, Evelyn R. Barrack, Sahn-Ho Kim, G. Prem-Veer Reddy

**Affiliations:** Vattikuti Urology Institute, Henry Ford Hospital, Detroit, Michigan, United States of America; II Università di Napoli, Italy

## Abstract

**Background:**

The androgen receptor (AR) plays a critical role in the proliferation of prostate cancer cells. However, its mechanism of action in proliferation remains unknown. An understanding of the mechanism of AR action in proliferation may lead to the development of effective strategies for the treatment of prostate cancer.

**Methodology/Principal Findings:**

In this study we report that pulse treatment of synchronized LNCaP cells with Casodex, an AR-antagonist, for 4 hours in mid-G_1_ phase was sufficient to prevent cells from entering S phase. Since the assembly of pre-replication complex (pre-RC) in G_1_ is required for the progression of cells from G_1_ to S phase, the effect of Casodex during mid-G_1_ suggested that the role of AR in proliferation might be to regulate the assembly of pre-RC. To test this possibility, we investigated the interaction between AR and Cdc6, an essential component of pre-RC in LNCaP cells. AR co-localized and co-immunoprecipitated with Cdc6, and Casodex treatment disrupted this interaction. AR-immunoprecipitate (AR-IP) also contained cyclin E and cyclin A, which play a critical role in pre-RC assembly and cell cycle entry into S phase, and DNA polymerase-α, PCNA, and ribonucleotide reductase, which are essential for the initiation of DNA synthesis. In addition, in cells in S phase, AR co-sedimented with components of the DNA replication machinery of cells that entered S phase.

**Conclusions/Significance:**

Together, these observations suggest a novel role of AR as a component of the pre-RC to exert control over progression of LNCaP cells from G_1_ to S phase through a mechanism that is independent of its role as a transcription factor.

## Introduction

Prostate cancer is the most frequently diagnosed non-skin cancer and second leading cause of cancer deaths in American men [1]. Androgen, by activating androgen receptor (AR), plays an important role in both the development and progression of prostate cancer. Hence, androgen ablation by pharmacological or surgical castration [2] remains frontline therapy for the treatment of locally advanced or disseminated prostate cancer. However, although the disease initially regresses in response to androgen ablation, it eventually relapses to become castration resistant [3], [4]. Remarkably however, despite the continued use of this treatment strategy for over 70 years, very little is known about the mechanism by which AR regulates prostate cancer cell proliferation. An understanding of the mechanism of AR action in proliferation may lead to the development of more effective strategies for the treatment of prostate cancer.

Castration-resistant growth of prostate cancer is often associated with increased expression of AR [5], [6], [7]. AR remains indispensable for the proliferation of prostate cancer cells that have become castrate-resistant; AR-specific shRNA or siRNA blocks proliferation of androgen-sensitive as well as castration-resistant AR-positive prostate cancer cells [8], [9], [10], [11], [12]. In addition, microinjection of AR-specific antibody into the nucleus or treatment of cells with AR mRNA hammerhead ribozyme inhibits the proliferation of AR-positive, but not AR-negative, prostate cancer cells [13], indicating a critical role of AR in proliferation of AR-expressing prostate cancer cells. Interestingly, the role of AR in proliferation of prostate cancer cells seems to be independent of its function as a transcription factor, at least in castrate resistant CWR22R3 human prostate cancer cells (derived from the CWR22 xenografts), in which AR-dependent proliferation is ligand independent but AR transcriptional activity remains ligand-dependent [11]. Collectively, these observations invoke the possibility that, besides its role as a transcription factor, AR may play a direct role in cell cycle regulatory events required for proliferation of prostate cancer cells. A direct role of AR in regulating proliferation contrasts with the notion of an indirect role in which AR transcriptional activity regulates the expression of factors required for cell cycle progression.

Cell cycle progression from G_1_ to S phase is fundamental to proliferation. We reported previously that Casodex (bicalutamide), a specific inhibitor of AR, blocks the ability of G_1_ phase AR-positive LNCaP prostate cancer cells to enter S phase [14], [15], indicating a role of AR in cell cycle progression from G_1_ to S phase. Progression of cells from G_1_ to S phase requires a cascade of sequential events in G_1_ phase that contribute to the assembly of the DNA replication machinery in cells that enter S phase. These events include a) loading of Cdc6, replication licensing factor RLF-B/Cdt1, and mini-chromosome maintenance (Mcm) proteins onto the origin recognition complex (ORC) to form pre-replication complex (pre-RC), and b) unwinding of DNA by helicase (Cdc45) associated with pre-RC, and the assembly of enzymes of DNA synthesis to form mega-complexes required for initiation of DNA synthesis at the origins of DNA replication (see Reddy et al [16] for a review).

In this study, we investigated whether AR interacts with the components of pre-RC and DNA replication machinery. Our studies demonstrate for the first time that AR is required in mid-G_1_ phase, at the time when pre-RC assembly is known to begin, in order for LNCaP cells to enter S phase, and that AR is associated with cell cycle regulatory proteins and enzymes of DNA synthesis in a multienzyme complex isolated from S phase cells. These observations suggest AR involvement in proliferation through its interaction with cell cycle regulatory proteins and enzymes of DNA synthesis required for the onset of DNA synthesis and the progression of LNCaP cells from G_1_ to S phase.

## Materials and Methods

### Cell Culture

LNCaP cells were maintained in RPMI medium (Gibco BRL, Rockville, MD) containing 10% fetal calf serum (FCS), 2.5 mM glutamine, 100 µg/ml streptomycin, and 100 U/ml penicillin (complete medium) in a humidified incubator with 5% CO_2_ and 95% air at 37°C.

### RNA Isolation, RT-PCR, and Real-time RT-PCR (qRT-PCR)

RNA was isolated from control and Casodex treated cells and subjected to RT-PCR to determine PSA and GAPDH expression as described previously [14]. The primer sequences used for PSA are: 5′-gcacccggagagctgtgt (forward) and 5-gatcacgcttttgttcctgat (reverse) primers, and for GAPDH are 5′-gagatccctccaaaatcaagtg (forward) and 5′-ccttccacgataccaaagttgt (reverse). GAPDH was used as an internal control. The densitometry of the RT-PCR bands was performed as described previously [14]. qRT-PCR was performed on an Applied Biosystems 7500 Fast Real-Time PCR System (Applied Biosystems, Foster City, CA) by using Taqman gene expression assays for PSA (Assay Id: Hs02576345_m1) and 18S RNA (Assay Id: Hs03928985_g1) as described previously [17]. The relative level of PSA expression was quantified by using comparative ΔΔC_t_ with 18S RNA as internal control.

### Synchronization of LNCaP Cells by Isoleucine Deprivation

Isoleucine deprivation was carried out by our previously published method [15]. Cells were grown to 60% to 70% confluence, and then the medium was replaced with isoleucine-free RPMI 1640 (Life Technologies, Carlsbad, CA) supplemented with 6% dialyzed FCS (Life Technologies, Carlsbad, CA), 2.5 mM glutamine, 100 µg/ml streptomycin, and 100 units/ml penicillin. Cells were maintained in isoleucine-free medium for 36 hours in an incubator as above. Cells were released from isoleucine-block by replacing the medium with the complete medium containing 10% FCS and treated with Casodex (bicalutamide, gift from Astra Zeneca, England, UK) as indicated.

Since entry of cells into S phase is marked by the onset of DNA synthesis, the progression of synchronized cells from G_1_ to S phase was monitored by determining the ability of cells to incorporate ^3^H-thymidine into DNA as described previously [14], [15]. At regular intervals after release from isoleucine-block, cells were pulse-labeled with 2 µCi/ml ^3^H-thymidine (ICN Biomedicals, Inc., Costa Mesa, CA) for 30 minutes at 37°C in a humidified incubator. The radioactivity incorporated into acid-precipitable material was then determined as described [15].

### Preparation of Cell Extracts and Immunoprecipitation

Exponentially growing LNCaP cells harvested by scraping into phosphate-buffered saline (PBS) were pelleted by centrifugation for 10 minutes at 4°C at 2,000 rpm in a Sorvall RT7 centrifuge and resuspended in immunoprecipitation (IP) buffer (50 mM Tris-HCl, pH 7.4, 0.1% Triton X-100, 5 mM EDTA, 250 mM NaCl, 2 mM CaCl_2_, 50 mM NaF, and 0.1 mM Na_3_VO_4_) supplemented with protease inhibitor cocktail (P-8340, Sigma Chemical Co., St Louis, MO) at a density of approximately 1×10^7^ cells/ml. The cell suspension was then subjected twice to 30 pulses of sonication using a Branson Sonifier 250 (Branson Sonic Power Co., Danbury, CT), set at an output control of 2 and a duty cycle of 20, with intermittent cooling on ice. The sonicated cell extract was cleared by centrifugation in an Eppendorf Centrifuge 5415R at 6,000 rpm for 5 minutes at 4°C. Protein concentration in cleared extracts was assessed using BioRad Protein Assay reagent (Bio-Rad Laboratories, Hercules, CA).

For immunoprecipitation, cell extracts were diluted 5-fold in IP buffer supplemented with protease inhibitor cocktail and incubated at 4°C overnight with 4 µg/ml anti-AR antibodies (AR-N20 or AR-441) or anti-Cdc6 antibodies (H-304) (Santa Cruz Biotech, Santa Cruz, CA). Immune complexes were then adsorbed to Pierce Protein A/G Agarose beads (Thermo Scientific, Philadelphia, PA) for 2 hours at 4°C with gentle agitation. The adsorbed complexes were washed three times with IP buffer by centrifugation in an Eppendorf Centrifuge 5415R at 6,000 rpm for 5 minutes at 4°C, and then eluted with PAGE loading buffer (Bio-Rad, Richmond, CA). Control immunoprecipitates were prepared by using 4 µg/ml purified mouse or rabbit IgG (Antibodies Incorporated, Davis, CA) in place of anti-AR or anti-Cdc6 antibodies.

### Isolation of DNA Replication Complex Fraction from Nuclear Lysate

Synchronized LNCaP cells in G_1_ phase (1 hour after release from isoleucine-block) or in S phase (24 hours after release from isoleucine-block) were harvested and the nuclear lysate was prepared by a slight modification of the method of Subramanyam et al [18]. Harvested cells were pelleted by centrifugation for 10 minutes at 4°C at 2,000 rpm in a Sorvall RT7 centrifuge and resuspended in buffer A [0.16 M sucrose, 50 mM Tris-HCl (pH 7.6), 25 mM KCl, 10 mM MgCl_2_, 70 mM HEPES (pH 7.6), 0.025 mM CaCl_2_, 1 mM phenylmethylsulfonyl fluoride (PMSF), 2 mM DTT, 1 mM EDTA] at a density of 2×10^7^ cells/ml. The cell suspension was then homogenized in a motor-driven Wheaton homogenizer (Wheaton Co., Wheaton, IL) until ∼90% of the cells were stained with trypan blue dye (usually three strokes at a rotation setting of 3). The cytosolic supernatant was then separated from the nuclear pellet by centrifugation in an Eppendorf Centrifuge 5415R at 6,000 rpm for 5 minutes at 4°C. The nuclear pellet was suspended in a volume of buffer A equal to that used for homogenization and subjected twice to 30 pulses of sonication with a Branson Sonifier 250 (Branson Co., Danbury, CT) set at an output control of 2 and a duty cycle of 20, with intermittent cooling on ice. The sonicated nuclear extract was cleared by centrifugation in an Eppendorf Centrifuge 5415R at 6,000 rpm for 5 minutes at 4°C. The DNA replication complex fraction was then isolated by subjecting the nuclear lysate to sucrose density gradient centrifugation essentially as described by Reddy and Pardee [19]. Nuclear lysate (0.5 ml) was layered over a 4.8 ml linear gradient of 20–40% (wt/vol) sucrose in buffer A, which in turn was layered over a 66% sucrose pad (0.5 ml) and centrifuged in a Beckman SW 50.1 rotor at 4°C for 16 hours at 35,000 rpm. The gradient was then resolved into 0.5 ml fractions and the rapidly sedimenting fraction that contained DNA polymerase activity is referred to as the replitase complex fraction as described by Murthy and Reddy [20]. The complex fraction from G_1_ or S phase cells was then subjected to Western blot analysis to assess the presence of enzymes of DNA synthesis and cell cycle regulatory proteins.

### Western Blot Analysis

Samples were dissolved in PAGE loading buffer (Bio-Rad, Richmond, CA) and subjected to denaturing 10% polyacrylamide gel electrophoresis (SDS–PAGE) and then transferred to nitrocellulose membranes. Individual membranes were probed with antibodies against AR (AR-N20 or AR-441), Cdc6 (H-304), prostate specific antigen (PSA, C-19), Lamin B, DNA polymerase-α (STK1), PCNA (PC10), ribonucleotide reductase (R2, E-16), cyclin A (H-432), cyclin E (C-19) (all from Santa Cruz Biotech, Santa Cruz, CA), p27^Kip-1^, cyclin B (BD Transduction Lab, San Jose, CA), caspase 3 (Cell Signalling, Danvers, MA), or GAPDH (Chemicon, Temecula, CA). Immunoreactive bands were developed using horseradish peroxidase-conjugated secondary antibodies and SuperSignal WestPico chemiluminescent substrate (Pierce, Rockfold, IL) and visualized using X-ray film.

### Immunofluorescent Staining and Confocal Microscopy

LNCaP cells grown on glass slides were washed once with PBS, followed by fixation in 3.7% (vol/vol) formaldehyde for 20 minutes at 22°C. Cells were permeabilized in 0.5% (vol/vol) Triton X-100 for 15 minutes and blocked for 1 hour in 2% (wt/vol) BSA at 22°C. Slides were then incubated for 1 hour at 22°C with antibodies against AR (AR-N20 or AR-441) and Cdc6 or DNA polymerase-α followed by both goat-anti-mouse-fluorescein isothiocyanate (FITC)- and goat-anti-rabbit-tetramethylrhodamine B isothiocyanate (TRITC)-labeled secondary antibodies (Sigma-Aldrich, St. Louis, MO). After four washes with PBS, slides were mounted with Aqua-Poly/Mount (Polysciences Inc., Warrington, PA). Confocal laser scanning microscopy was performed with a Zeiss LSM 410 upright confocal microscope. Images were processed, and colocalization analysis (which is colored yellow) was performed with a Zeiss LSM 410 Meta Excitation system with collection at 488 and 543 nm. Intensity Correlation Quotient (ICQ) analysis to quantify AR colocalization with Cdc6 or DNA polymerase-α was performed by using ImageJ software (NIH, Bethesda, MD).

### Immunofluorescent Detection of Bromodeoxyuridine (BrdU) Incorporated into DNA

LNCaP cells grown on glass slides were labeled with 10 µM BrdU (Sigma-Aldrich, St. Louis, MO) for 30 minutes, washed once with PBS and fixed in 3.7% formaldehyde for 20 min at 22°C. Cells were permeabilized in 0.5% (v/v) Triton X-100 for 15 min and blocked for 1 hour in 2% (w/v) bovine serum albumin at 22°C. Cells were then stained with mouse monoclonal antibodies against BrdU (Sigma-Aldrich, St. Louis, MO), followed by goat-anti-mouse-FITC-labeled secondary antibody. Slides were mounted with Vectashield Mounting Medium containing DAPI (Vector Laboratories, Burlingame, CA) and photomicrography was performed with a Zeiss Axiofot microscope equipped for epifluorescence.

## Results

### Use of Casodex to Determine the Role of AR in the Cell Cycle of Synchronized LNCaP Cells

AR knockdown strategies have been used to demonstrate that AR is required for proliferation of prostate cancer cells [8], [9], [10], [11], [12]. However, AR knockdown by siRNA or shRNA requires treatment of prostate cancer cells over a period of several days. Thus, this approach can not be used to identify when in the cell cycle (i.e., G_1_, S, or G_2_/M phases) AR is required for proliferation of LNCaP cells, as each phase of the cell cycle lasts no more than few hours. Therefore, we used the non-steroidal anti-androgen Casodex (bicalutamide) to selectively and acutely inhibit AR in order to determine the role of AR in progression of synchronized LNCaP cells through G_1_ and S phases.

**Figure 1:**
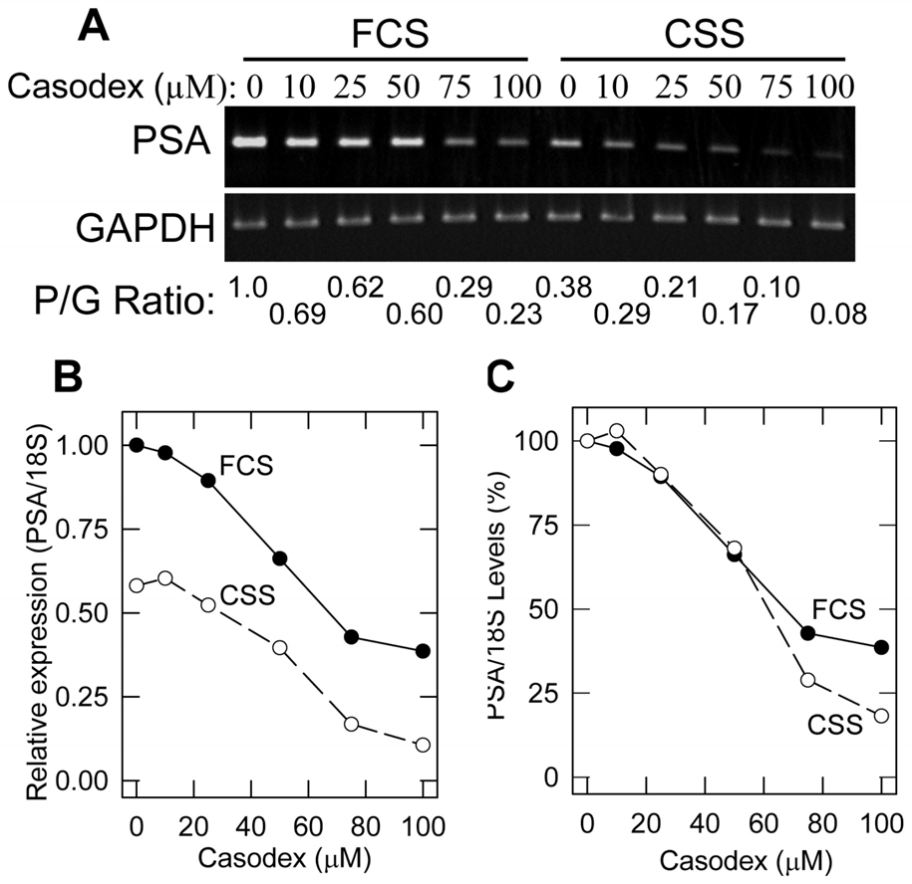
Casodex dose-response inhibition of LNCaP cell AR activity in FCS- vs. CSS- containing medium. Exponentially growing LNCaP cells were washed twice with serum- and hormone- free medium and transferred to RPMI medium containing 10% fetal calf serum (FCS) or 6% charcoal-stripped serum (CSS). After 24 hours, Casodex was added and cells were incubated for an additional 24 hours. RNA isolated from control and Casodex treated cells was subjected to RT-PCR and qRT-PCR analysis of PSA as described in Materials and Methods. (A) RT-PCR products of PSA and GAPDH separated on 1% agarose gel. The ratio of band density PSA/GAPDH (P/G ratio) of cells in FCS without Casodex was set at 1.0 and the P/G ratio of other treatment conditions is expressed relative to this control. (B) Relative PSA levels, as determined by qRT-PCR, are plotted as a function of Casodex concentration. And (C) Relative PSA levels in Fig. 1B are plotted as a percentage of control without Casodex calculated separately for FCS- and CSS- medium. Closed circle, FCS-medium; and open circles, CSS-medium. The data is representative of two independent experiments.

Casodex is usually used at 10 to 20 µM to inhibit androgen-induced AR transcriptional activity; but in those experiments, the cells are in charcoal-stripped serum (CSS)-containing medium [21], [22]. However, synchronized cells released into CSS-containing medium will not progress through the cell cycle. We compared the effectiveness of Casodex on the expression of prostate specific antigen (PSA), a specific AR-target gene [23], in LNCaP cells in FCS- vs. CSS- medium. Androgen-deprivation of cells in CSS-medium decreased PSA expression to ∼50% of that in control cells in FCS-medium and Casodex caused a further decrease (Figs. 1A and 1B). Casodex at 75–100 µM in FCS-medium had the same effect on PSA expression as 25–50 µM Casodex in CSS-medium (Figs. 1A and 1B). Notably, when the dose-dependent response to Casodex is plotted relative to its own control (in FCS vs. CSS), the curves overlap, indicating that the IC_50_ to inhibit AR activity is ≈50 µM Casodex in either FCS or CSS (Fig. 1C). Cells treated with 100 µM Casodex in FCS-medium were morphologically similar to those treated with 20 µM Casodex in CSS-medium, and 100 µM Casodex in FCS-medium caused no noticeable cell death, as determined by the Trypan Blue exclusion method (data not shown). Therefore, we used 100 µM Casodex, a maximally effective concentration to inhibit AR activity, to determine the role of AR in proliferation of LNCaP cells in FCS-medium. This concentration is similar to that used to inhibit PSA expression or growth of LNCaP cells in the presence of FCS [24], [25], [26].

### LNCaP Cell Entry into S Phase Requires Functional AR during Mid-G_1_ Phase

**Figure 2:**
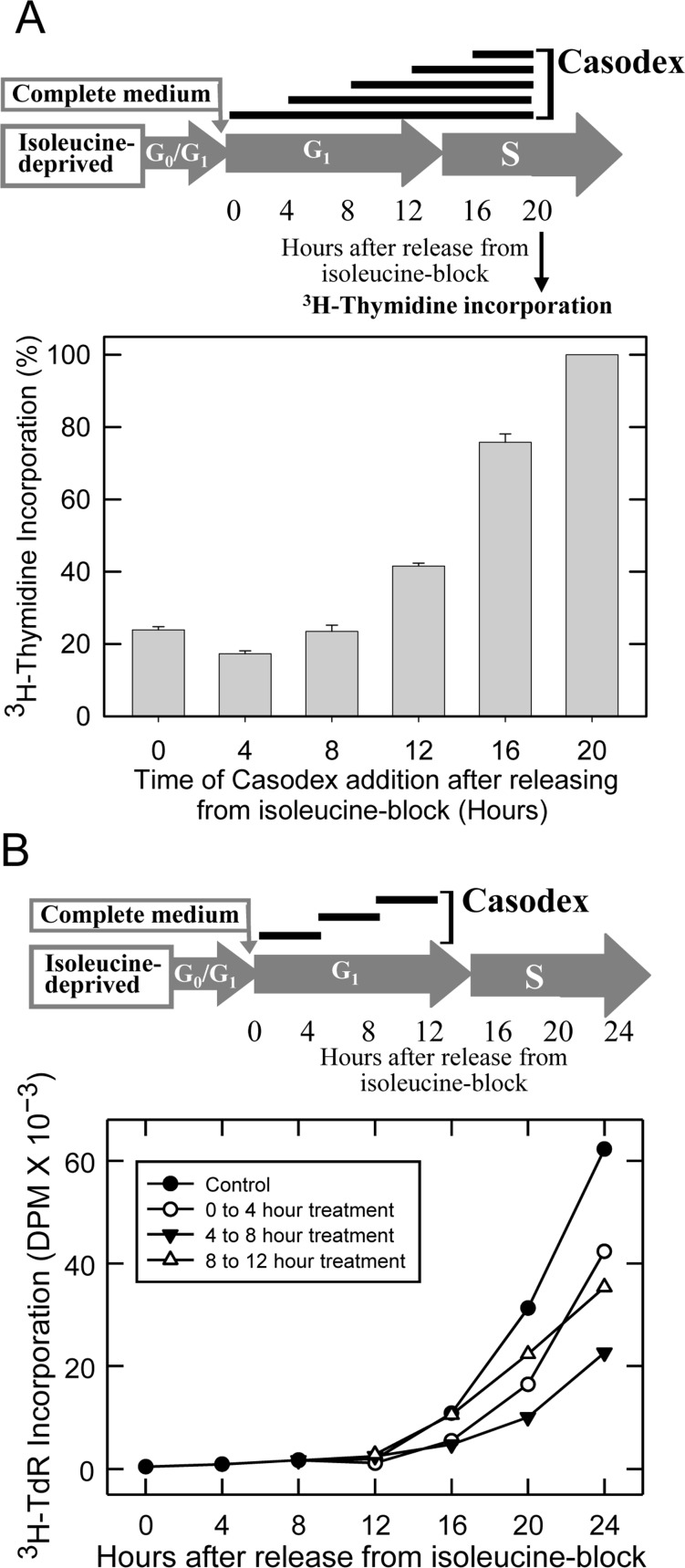
Casodex inhibits the progression of synchronized LNCaP cells from G_1_ to S phase: LNCaP cells were synchronized by isoleucine-deprivation and released into complete medium. A) Casodex was added at the indicated time after release from isoleucine-block and maintained in the medium until ^3^H-TdR incorporation was determined at 20 hours after release from isoleucine-block. Results are expressed as percentage of ^3^H-TdR incorporation in control cells that were released into complete medium in the absence of Casodex. A) Casodex was added for a 4-hour period as shown in the top panel, and ^3^H-thymidine (^3^H-TdR) incorporation into DNA was determined every 4 hours after release from isoleucine-block and after removal of Casodex. Each data point is the average of triplicate samples with <10% variation; data shown are representative of two independent experiments.

We have shown previously that isoleucine deprivation causes LNCaP cells to arrest in G_0_/G_1_ phase [15], [27]. Upon release from isoleucine-block, i.e., transfer into complete medium containing FCS (FCS-medium), these cells progress through G_1_ and enter S phase 12 hours after release, and reach a peak of S phase 10–12 hours thereafter [15]. We have also shown that Casodex treatment for 24 hours starting from the time of release from isoleucine-block abrogates the ability of cells to enter S phase [14], [15]. While this suggested a role of AR in the cell cycle, that experimental design cannot distinguish between a role of AR in G_1_ vs. S phase. Therefore, in order to distinguish between an inhibitory effect of Casodex in G_1_ vs. in S phase, we treated synchronized LNCaP cells with Casodex starting at 0, 4, 8, 12, or 16 hours after release from isoleucine-block, and determined the ability of cells to incorporate ^3^H-thymidine (^3^H-TdR) at 20 hours after release, a time when control cells are in S phase (see schematic of Fig. 2A). As shown in Fig. 2A, Casodex maximally inhibited ^3^H-TdR incorporation if added any time during G_1_ phase (i.e., 0, 4, or 8 hours after release), indicating a role of AR in G_1_. By contrast, the Casodex effect was blunted if treatment was delayed until 12–16 hours after release from isoleucine-block, a time when the cells have already committed to enter S phase. Casodex treatment of cells that had already entered S phase (16 hours after release from isoleucine-block) had very little effect on ^3^H-TdR incorporation (Fig. 2A). Thus, the inhibitory effect of Casodex occurs in G_1_ phase, when cells are preparing to enter S phase, and not in S phase. Therefore, AR is required in G_1_ for cells to enter S phase.

We then determined when in G_1_ phase Casodex is most effective in suppressing cell entry into S phase. We pulse treated synchronized LNCaP cells with Casodex during the first, second, or third 4-hour interval following release from isoleucine-block, corresponding to early-G_1_, mid-G_1_, or late-G_1_, respectively, and determined the rate of ^3^H-TdR incorporation into DNA at 4-hour intervals for 24 hours (see schematic of Fig. 2B). We have shown previously that ^3^H-TdR incorporation offers a reliable and reproducible method to monitor the progression of synchronized cells from G_1_ to S phase and is corroborated by flow cytometry analysis as well as the expression of S phase-specific cyclins and the activation of cyclin-dependent kinases [14], [15]. As shown in Fig. 2B, treatment with Casodex during mid-G_1_ (4–8 hours after release from isoleucine-block) was more effective at blocking entry into S phase than was treatment during early-G_1_ (0–4 hours after release from isoleucine-block) or late-G_1_ phase (8–12 hours after release from isoleucine-block). This suggests AR involvement in cell cycle regulatory events in mid-G_1_ phase that are critical for LNCaP cells to progress from G_1_ to S.

### AR Interacts with Cdc6, a Critical Component of Pre-RC Required for the Assembly of Replication Machinery in S Phase LNCaP Cells

**Figure 3:**
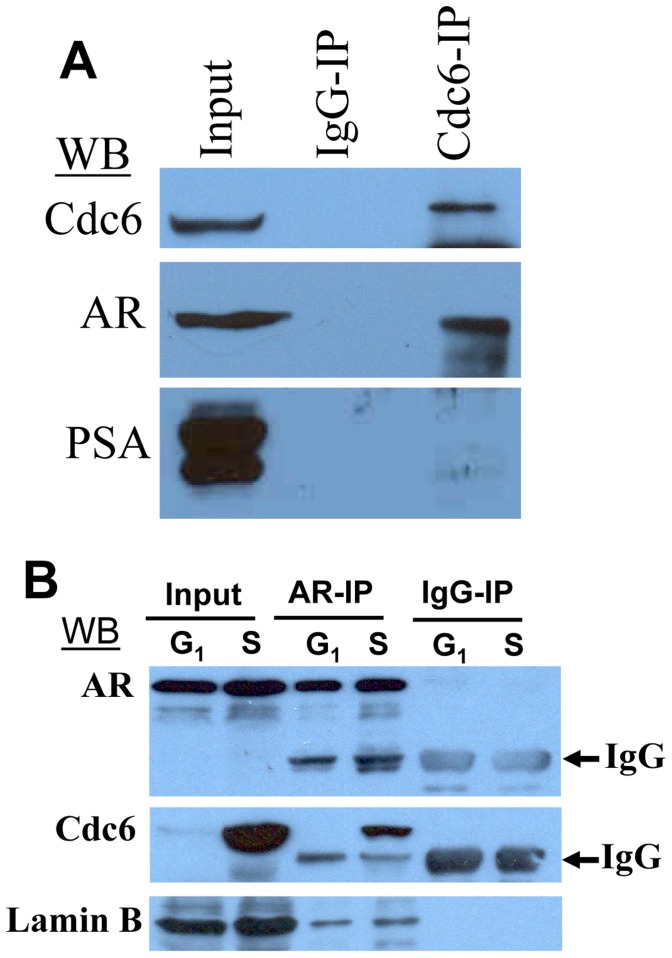
AR in LNCaP cell extracts co-immunoprecipitates with Cdc6: A) Cdc6-IP was prepared from exponentially growing LNCaP cells and was subjected to Western blot analysis. B) AR-IPs were prepared from synchronized G_1_ phase (1 hour after release from isoleucine-block) or S phase (20 hours after release from isoleucine-block) LNCaP cells, and subjected to Western blot analysis. IgG heavy chain in AR-IP and IgG-IP is indicated by arrow.

Since Casodex treatment in mid-G_1_ blocked the ability of synchronized LNCaP cells to enter S phase (Fig. 2), we tested whether AR interacts with cell cycle regulatory proteins involved in the assembly of pre-RC, as assembly of pre-RC in mid- to late-G1 is required for cells to enter S phase and initiate DNA synthesis. Cdc6 binding to the origin recognition complex (ORC) is the first step in the process of pre-RC assembly [16]. We observed that an immunoprecipitate prepared using Cdc6-specific antibodies (Cdc6-IP) contained AR, suggesting AR interaction with Cdc6 (Fig. 3A). This interaction is specific since one of the most abundantly expressed proteins, prostate-specific antigen (PSA), in LNCaP cells was not associated with Cdc6-IP (Fig. 3A). We have shown previously that the interaction between cell cycle regulatory proteins and enzymes of DNA synthesis in a variety of cells leads to the assembly of a multienzyme complex called replitase or DNA replication machinery [28], [29], [30]. These complexes are readily detectable in cells that are in S phase, but not any time during G1 phase [29], [30]. Therefore, we tested whether AR-Cdc6 interaction is a cell cycle-dependent phenomenon. As shown in Fig. 3B, although Cdc6 was present at a low level in G_1_ phase cells, its association with AR-IP was seen in synchronized LNCaP cells that were in S phase but not in those that were in G_1_ phase. By comparison, lamin B, a nuclear protein, showed very little difference in its association with AR-IP from G_1_ vs. S phase cells (Fig. 3B). Thus, AR exhibits a cell cycle-dependent interaction with Cdc6.

### Casodex Disrupts AR-Cdc6 Interaction

**Figure 4:**
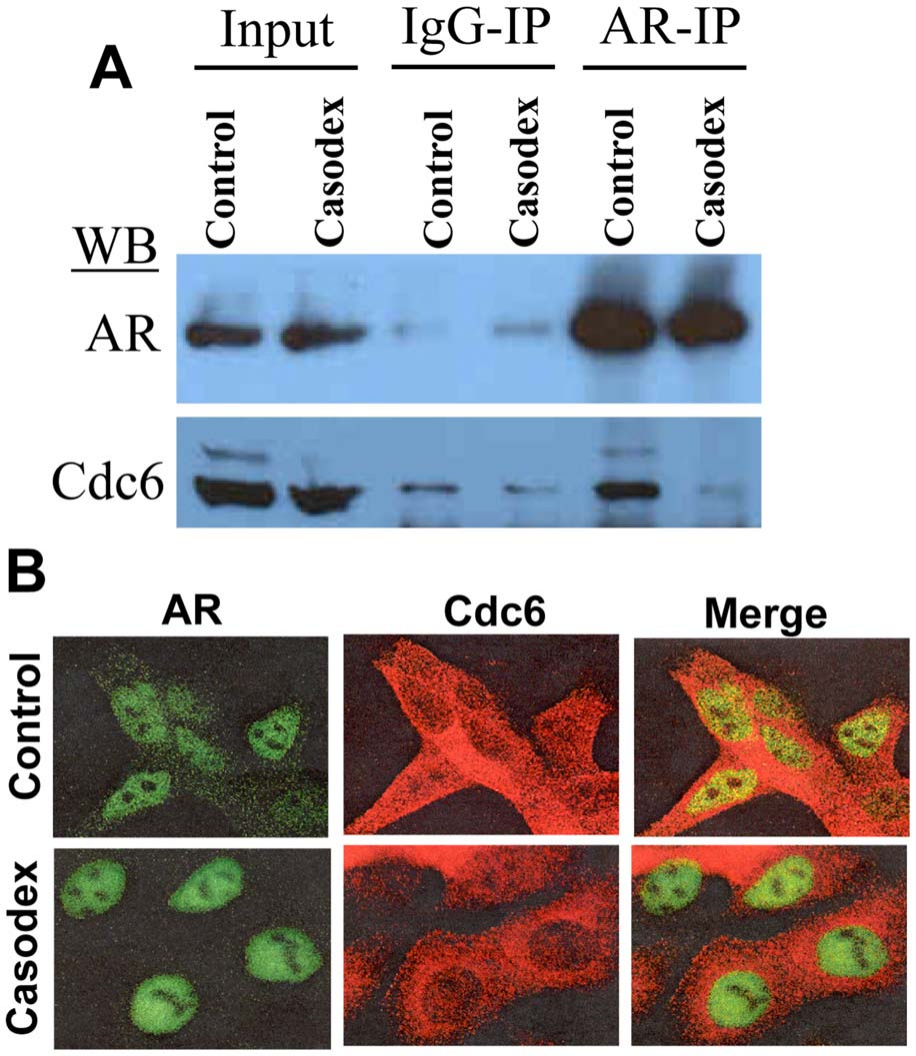
Casodex inhibits AR-Cdc6 interaction in LNCaP cells: A) Exponentially growing LNCaP cells were treated with Casodex or vehicle (control) for 24 hours and AR-IP prepared from treated and control cells was subjected to Western blot analysis to detect AR and Cdc6. B) LNCaP cells grown on slides were synchronized by isoleucine-deprivation and released into complete medium in the presence of vehicle (control) or Casodex. At 24 hours after release from isoleucine-block (when control cells are expected to enter S phase), cells were fixed and processed for immunoflourescent staining using anti-AR (AR-441) mouse monoclonal and anti-Cdc6 (H-304) rabbit polyclonal antibodies.

Since AR is implicated to play a role in regulation of Cdc6 expression [14], [31], we tested whether Casodex-induced blockade of the entry of LNCaP cells into S phase is due to a decrease in Cdc6 protein levels. In fact, under the experimental conditions employed in the present study, Casodex had no noticeable effect on either Cdc6 or AR protein levels in exponentially growing LNCaP cells (Fig. 4A, Input). Nonetheless, Casodex disrupted AR interaction with Cdc6 as indicated by the presence of Cdc6 in AR-IP of control cells but not in that of Casodex treated cells (Fig. 4A, AR-IP). This Casodex-induced disruption of AR association with Cdc6 was further supported by confocal microscopy (Fig. 4B); we observed a noticeable decrease in the colocalization of AR with Cdc6 in the nuclei of Casodex treated cells (ICQ = 0.059±0.022 SD) as compared to control cells (ICQ = 0.152±0.047 SD). Thus, Casodex-induced suppression of the entry of LNCaP cells into S phase is associated with the disruption of AR-Cdc6 interaction.

### AR Interacts with S Phase Cyclins

**Figure 5:**
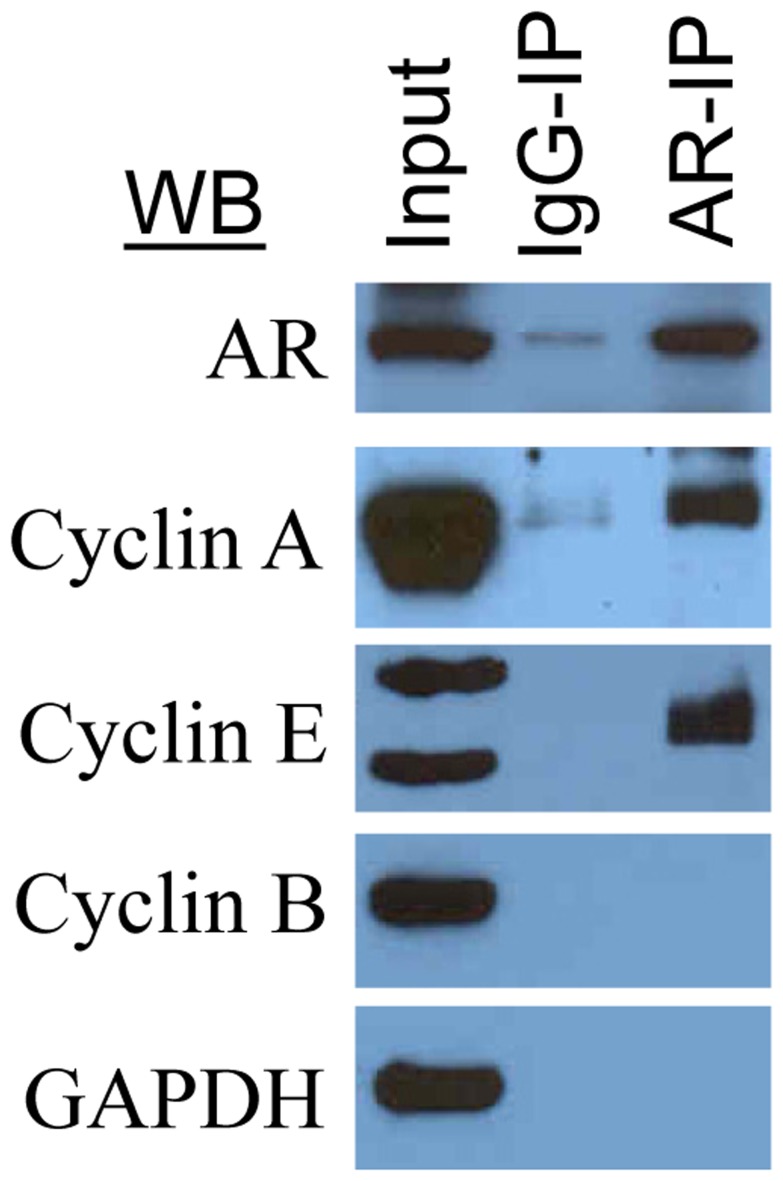
AR-IP contains cyclin A and cyclin E, but not cyclin B: AR-IP prepared from exponentially growing LNCaP cells was subjected to Western blot analysis.

Besides Cdc6, some of the cell cycle regulatory proteins, such as cyclins E and A, also play an important role in the assembly of pre-RC in G_1_ [16]. Therefore, we tested whether AR interacts with any of the cyclins that are known to be involved in the progression of cells from G_1_ to S phase (viz., cyclins E and A) and G_2_/M (viz., cyclin B) phases in LNCaP cells. We observed that cyclin E and cyclin A, but not a mitotic cyclin, cyclin B, were associated with AR-IP prepared from exponentially growing LNCaP cells (Fig. 5). By comparison, GAPDH, a cytosolic marker, was not associated with AR-IP. Thus, AR exhibits a specific interaction with cell cycle regulatory proteins that are involved in regulation of cell entry into S phase.

### AR Interacts with Enzymes of DNA Synthesis

**Figure 6:**
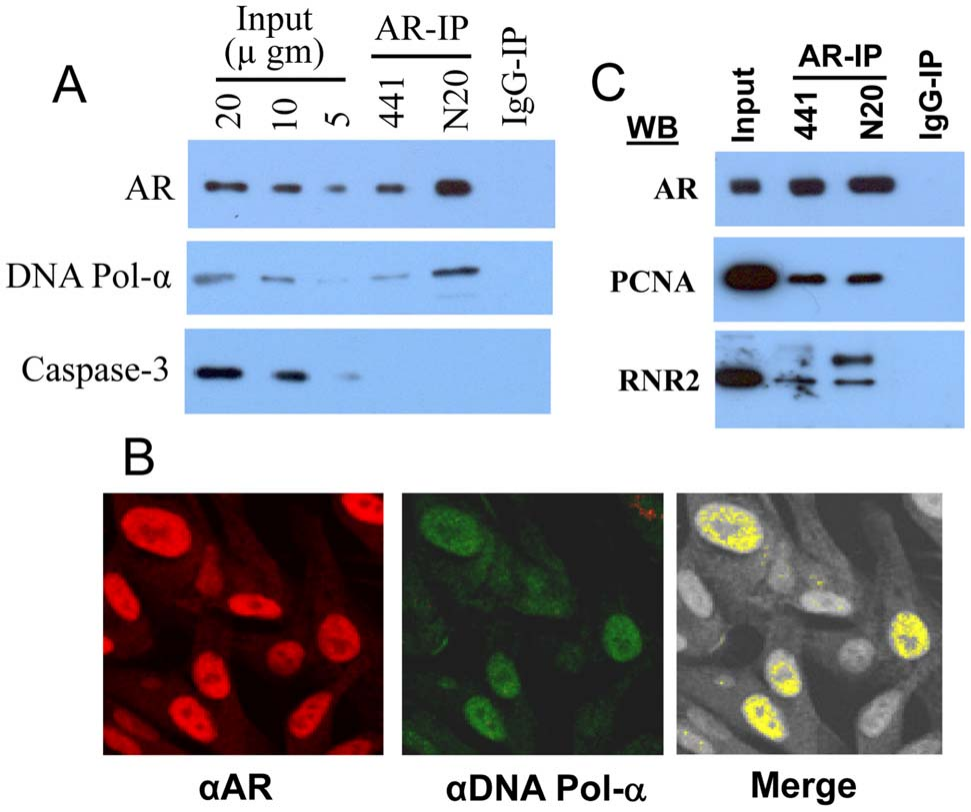
AR interacts with enzymes of DNA synthesis: A) AR-IP contains DNA polymerase-α. AR-IP prepared from exponentially growing LNCaP cells by using anti-AR mouse monoclonal (441) or rabbit polyclonal (N-20) antibodies was subjected to Western blot analysis. B) AR is colocalized with DNA polymerase-α in LNCaP cells. Exponentially growing LNCaP cells on slides were fixed and stained with anti-AR (N-20) rabbit polyclonal and anti-DNA polymerase-α (STK1) mouse monoclonal antibodies and confocal microscopy was performed. C) AR-IP contains PCNA and ribonucleotide reductase. AR-IP prepared from exponentially growing LNCaP cells was subjected to Western blot analysis. RNR2, ribonucleotide reductase catalytic subunit.

Since Cdc-6-dependent assembly of pre-RC leads to the recruitment of enzymes of DNA synthesis to the sites of DNA replication [16], and since AR interacts with Cdc6 (Fig. 3), we tested whether AR-IP also contains enzymes of DNA synthesis. As shown in Fig. 6, we observed that AR-IP prepared using two different AR antibodies (AR-N20 and AR-441) contained DNA polymerase-α, a key enzyme involved in initiation of DNA synthesis (Fig. 6A). AR interaction with DNA polymerase-α was further corroborated by immunoflourescent confocal microscopy (Fig. 6B); we observed a strong colocalization of AR with DNA polymerase-α (ICQ = 0.45±0.016 SD) in the nuclei of LNCaP cells. In addition to DNA polymerase-α, AR-IP prepared using AR-441 and AR-N20 antibodies contained proliferating cell nuclear antigen (PCNA), an auxiliary protein of DNA polymerase, and the catalytic subunit of ribonucleotide reductase (RNR2), which converts ribonucleotides to deoxyribonucleotides required for DNA synthesis (Fig. 6C). In addition to its role in proliferation [8], [9], [10], [11], [12], AR is also reported to play a critical role in survival of LNCaP cells, as AR knockdown leads to apoptotic cell death [32]. Therefore we tested whether the role of AR in regulation of apoptosis also involves its interaction with apoptotic proteins. As shown in Fig. 6A, caspase-3, an apoptotic enzyme, was not detected in AR-IPs. Thus, AR exhibits a selective interaction with the enzymes of DNA synthesis required for the proliferation of LNCaP cells.

### Enzymes of DNA Synthesis Co-sediment with AR in Nuclear Lysate of S, but not G_1_, Phase LNCaP Cells

**Figure 7:**
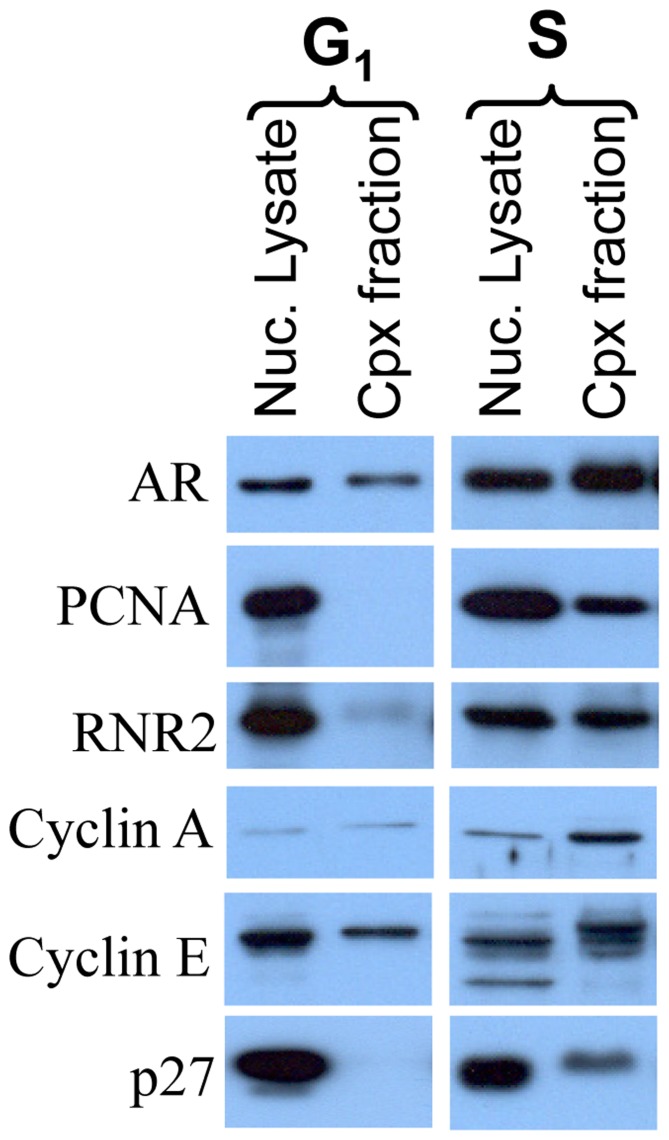
Differential association of AR with cell cycle regulatory proteins and enzymes of DNA synthesis in replitase complex from synchronized LNCaP cells in G_1_ vs. S phase: LNCaP cells were synchronized by isoleucine-deprivation and replitase complex (Cpx) fraction was prepared from the nuclear lysate of cells in G_1_ phase (1 hour after release from isoleucine-block) or S phase (20 hours after release from isoleucine-block). Equal amounts of protein were subjected to Western blot analysis. RNR2, ribonucleotide reductase (RNR2).

Progression of cells from G_1_ to S phase is associated with the assembly of enzymes of DNA synthesis into replitase complexes that can be isolated by biochemical fractionation of nuclear lysate [29], [30]. We tested whether the entry of LNCaP cells into S phase involves AR interaction with the enzymes of DNA synthesis in replitase complexes. We have shown previously that AR co-sediments with DNA polymerase activity in the replitase fraction isolated from exponentially growing LNCaP cells [20]. We now report that other enzymes of DNA synthesis, viz., PCNA and ribonucleotide reductase, and cell cycle regulatory proteins, viz., cyclin A, cyclin E and p27^Kip-1^, also co-sediment with AR (Fig. 7). Importantly, this co-sedimentation was seen in nuclear lysate prepared from cells that were in S phase (20 hours after release from isoleucine-block), but not G_1_ phase (1 hour after release from isoleucine block) (Fig. 7). Thus, there is an S phase-specific association of AR with enzymes of DNA synthesis in a replitase complex fraction.

## Discussion

The ability of cells to enter S phase is determined by events in G_1_ phase. While a role of AR in the control of proliferation of prostate cancer cells is evident from a number of studies that knocked down AR in androgen-sensitive and castration-resistant prostate cancer cells [8], [9], [10], [11], [12], the mechanism by which this occurs remains elusive. Our studies demonstrate for the first time that AR is required in mid-G_1_ phase for synchronized LNCaP cells to progress from G_1_ to S phase and that AR interacts with cell cycle regulatory proteins and enzymes required for initiation of DNA synthesis. While these observations remain to be validated in other AR-positive prostate cancer cell lines, the data presented here suggest AR involvement in proliferation of LNCaP cells through its interaction with components of the pre-RC necessary for the initiation of DNA synthesis and, therefore, for the progression of cells from G_1_ to S phase.

We observed that the anti-proliferative effect of Casodex is quite evident in LNCaP cells that were treated with Casodex specifically during mid-G_1_ phase but not in those that had already entered S phase (Fig. 2). Interestingly, this is consistent with an earlier report in which cyproterone acetate, another AR antagonist, blocks testosterone-stimulated DNA synthesis in the prostate gland of castrated rat when administered with testosterone but not if cyproterone acetate is delayed until DNA synthesis has already started [33].

**Figure 8:**
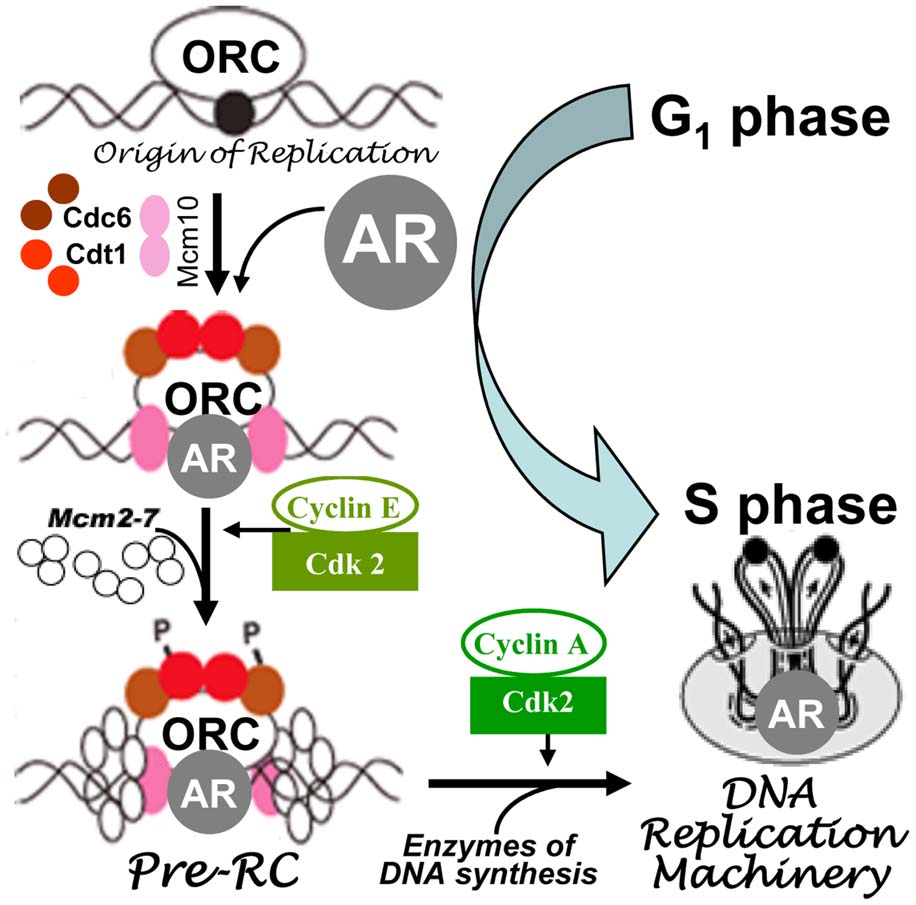
Model of AR interaction with pre-RC and replication machinery as cells progress from G_1_ to S phase. Pre-RC assembly involves sequential recruitment of Cdc6, Cdt1, and mini-chromosome maintenance (Mcm) proteins to the origin recognition complex (ORC), which serves as a “launching pad” for the assembly of DNA replication complexes/machinery at the origin of DNA replication. Cyclin-dependent kinases, such as Dbf4-Cdc7 kinase (not shown), cyclin E-Cdk-2 and cyclin A-Cdk-2, play a role in the recruitment of Mcm proteins and enzymes of DNA synthesis to form DNA replication machinery as cells progress from G_1_ to S phase. AR integrates into DNA replication machinery through its interaction with pre-RC components. Events occurring at one of the many origins of DNA replication that exist in a proliferating cell are depicted.

Our observations suggest a role of AR in events leading up to the transition of cells from G_1_ to S phase. Pre-RC assembly is one such event that occurs during that period and is necessary for the initiation of DNA synthesis and, therefore, for the progression of cells from G_1_ to S phase [16]. We observed that AR co-immunoprecipitates with Cdc6, a critical component of pre-RC (Fig. 3). There are other pre-RC components, such as Orc2, Cdt1, and Mcm7, that are also reported to be associated with AR in prostate cancer cells [34], [35]. Interestingly, Cdc6 association with AR-IP was abrogated in cells treated with Casodex (Fig. 4), indicating a critical role of AR-Cdc6 interaction in progression of LNCaP cells from G_1_ to S phase. Progression of cells from G_1_ to S phase requires not only pre-RC assembly but also the recruitment of enzymes of DNA synthesis to pre-RC. DNA synthesis requires the concerted action of a number of enzymes and proteins in megacomplexes, which are referred to as DNA replication machinery, DNA synthesome or replitase [36], [37], [38]. Pre-RC serves as a “launching pad” for the assembly of DNA replication machinery at sites of DNA replication [16]. Our studies revealed AR association with DNA replication machinery specifically in cells that entered S phase, but not in those that were in G_1_ phase (Fig. 7). AR integration into DNA replication machinery is also supported by the observation that AR is localized at sites of DNA replication [39]. Thus, AR integration into replication machinery through its interaction with the components of pre-RC as depicted in Fig. 8, may allow AR to exert control over proliferation of prostate cancer cells.

Based on the critical role of AR interaction with Cdc6 in cell cycle progression, which is indicated from the observation that Casodex disrupts AR interaction with Cdc6 (Fig. 4) and inhibits the progression of cells from G_1_ to S phase (Fig. 2), we propose that AR plays a direct role in proliferation, independent of its role in regulating the expression of genes required for proliferation. In addition, whereas a 4-hour Casodex treatment was sufficient to block G_1_ phase cells from entering S phase (Fig. 2), a similar 4-hour treatment with Casodex had no noticeable effect on AR transcriptional activity, as determined by PSA mRNA expression, in LNCaP cells (data not shown). Thus, the Casodex-sensitive role of AR in progression of cells from G_1_ to S phase, i.e., proliferation, seems to be independent of its role as a transcription factor. The AR indeed has been shown to exhibit a critical function in proliferation of prostate cancer cells that is distinct from its function as a transcription factor; in CWR22R3 cells, the AR role in proliferation, but not its role as a transcription factor, is androgen-independent [11]. Similarly, Sathya et al [40] showed that the regulation of AR transcriptional activity and the role of AR in proliferation are mechanistically distinct; they developed tissue-specific AR modulators (SARMs) that are very weak as activators of AR transcriptional activity but are as effective as dihydrotestosterone (DHT) in stimulating the proliferation of prostate cancer cells.

Since cells that lack AR can progress flawlessly through the entire cell cycle, it seems remarkable that AR would integrate into the replication machinery to regulate proliferation of AR-positive prostate cancer cells. Interestingly, AR is not the only transcription factor that exerts control over proliferation through integration into the replication machinery. E2F-1 and Rb protein, both of which regulate the expression of genes necessary for cell cycle progression and DNA replication [41], [42], also function at replication origins to limit DNA replication by interacting with proteins of the ORC, not by acting as transcription factors [43], [44]. Conversely, proteins involved in the assembly of pre-RC, viz., Mcm proteins 2–7, colocalize with RNA polymerase II on actively transcribing genes and are required for transcription elongation [45]. These interactions between transcription factors and proteins involved in the assembly of pre-RC may facilitate the orderly replication of transcriptionally active regions of genomic DNA in S phase. Interestingly, the timing of initiation of replication in a genomic region is dictated by the transcriptional activity in that region [46], [47]; transcriptionally active regions are known to replicate early in S phase, whereas transcriptionally inactive regions replicate late in S phase [48], [49]. Furthermore, origins of DNA replication are enriched around transcription start sites and transcription initiation is reported to influence the initiation of DNA replication [50], [51]. Thus, there is a cross-talk between transcription and replication, which may be facilitated by the interactions between proteins involved in DNA replication and transcription. Therefore, we propose that AR associated with proteins and enzymes of pre-RC and replication machinery may play a role in coordinating the replication of transcriptionally active AR-target genes during S phase.

In summary, AR may exert control over proliferation of prostate cancer cells through its interaction with pre-RC and DNA replication machinery. Future studies focusing on identification of protein(s) that interface with AR in pre-RC may lead to the development of new and more effective strategies to suppress AR-dependent growth of prostate cancer. In addition, altered interaction of AR with pre-RC and replication machinery could circumvent the requirement for androgen and account for the continued critical role of AR in proliferation of castration-resistant prostate cancer cells. Therefore, analysis of pre-RC and replication machinery constituents in androgen-sensitive vs. castration-resistant prostate cancer cells may yield valuable information about the mechanism of AR action in prostate cancer cell proliferation and may lead to the identification of potentially more effective therapeutic targets for prostate cancer treatment.
